# AI literacy and school tier moderate educators’ ethical coping mechanisms through sedative and reverse Matthew effects

**DOI:** 10.1038/s41598-026-52376-z

**Published:** 2026-05-16

**Authors:** Jun Wang, Yang Hu, Huilin Tang

**Affiliations:** 1 Faculty of Humanities and Social Sciences, City University of Macau, Macau SAR, China; 2 School of Foreign Languages and Foreign Trade, Guilin University of Aerospace Technology, Guilin, China; 3 Nanning Vocational and Technical University, Nanning, China

**Keywords:** AI ethics, Dual-dimension risk–coping framework, AI literacy, Reverse Matthew Effect, Educators, PLS-SEM, Education, Information systems and information technology, Psychology, Psychology, Science, technology and society

## Abstract

The rapid penetration of artificial intelligence in education has triggered unprecedented ethical risk perception and response challenges for educators, but existing research lacks systematic exploration of deep ethical cognitive mechanisms. To address this, this study operationalizes macro level sociotechnical imaginaries into micro level psychological perceptions to construct and test a theoretical model that integrates dual-dimensional risk perception, AI Literacy, and situational factors. PLS-SEM analysis of 502 Chinese educators reveals that: both Inhibitory Ethical Risk and Promotional Professional Risk are significantly and positively associated with Defensive and Constructive Coping strategies simultaneously, challenging the traditional one dimensional risk hypothesis; AI Literacy significantly attenuated the positive association between Inhibitory Ethical Risk and Constructive Coping, supporting the hypothesis of the Literacy Sedative Effect; The strength of the positive association between Promotional Professional Risk and Constructive Coping in lower tier schools is 2.78 times stronger than that in first tier schools, providing empirical evidence for the Reverse Matthew Effect. This study proposes a dual-dimensional risk-coping framework, revealing the cognitive decoupling mechanism of AI Literacy and challenging the classical Matthew Effect, providing a new perspective for understanding individual behavior in disruptive technological change; at the same time, it also provides practical guidance for cultivating cautious and proactive adopters and developing differentiated AI Literacy training strategies.

**Supplementary Information:**

The online version contains supplementary material available at 10.1038/s41598-026-52376-z.

## Introduction

Artificial intelligence (AI), especially generative AI, has triggered unprecedented cognitive challenges. Major economies show strategic differentiation: China and the United States are accelerating efforts to seize leadership positions^1,2^,while the European Union prioritizes ethics and security through the Artificial Intelligence Act^3^. This tension between development and regulation reflects different sociotechnical imaginaries of an ideal future^4^. AI is rapidly infiltrating society, with a surge in global private investment^5^, particularly in education. However, this has also raised serious ethical challenges ranging from algorithmic bias^6^to data trust^5^. Educators are internalizing this macro dilemma as a psychological conflict, making their risk perception and coping mechanisms a key academic issue.

Although the transactional stress and coping model^7^provides a foundational framework for understanding how individuals evaluate environmental demands (e.g., inhibitory ethical and promotional professional risks) and respond to them (e.g., via defensive or constructive coping), its application to AI contexts exposes three theoretical puzzles. First, the paradox of parallel activation: why are AI risks simultaneously associated with two strategies that typically compete for cognitive resources? Second, the ambiguous role of AI Literacy: why does higher AI Literacy, a key capability beyond the classical Technological Pedagogical Content Knowledge (TPACK) framework^8,9^, seem to negatively moderate rather than strengthen the relationship between risk and constructive action? Third, the contextual paradox of resource allocation: why might resource poor environments exhibit stronger innovation efficiency (contrary to the classical Matthew Effect^10^, challenging the conventional digital divide theory by highlighting technological agency^11,12^?

To address these gaps, this study operationalizes macro level sociotechnical visions into micro level psychological perceptions to examine three core questions: (1) How are Inhibitory Ethical and Promotional Professional Risks related to both Defensive and Constructive Coping in parallel? (2) How does AI Literacy moderate the Inhibitory Ethical Risk to Constructive Coping pathway? (3) Does School Tier moderate the Promotional Professional Risk to Constructive Coping pathway?

## Theoretical framework

### Dual AI risks and differentiated coping

Although research on the adoption of generative AI in education has proliferated^13^, and a unified model integrating internal and external determinants has emerged^14^, most discussions remain limited to the instrumental dimensions of traditional technology acceptance models^15^,failing to address ethical and cognitive mechanisms^16,17^. Adopting a sociotechnical perspective (e.g., Actor Network Theory^18^, we view technology adoption as a social construction process that goes beyond isolated individual decision making. Sociotechnical Imaginaries^4^, which posit that collective visions of desirable futures shape technological responses, are drawn upon to inform this view. These macro imaginaries are operationalized at the micro psychological level via cognitive appraisal mechanisms from Transactional Stress Theory^7^, translating macro threats and opportunities into two individual perceptions: the progressive imagination of opportunity^19^, termed Promotional Professional Risk Perception (PP), and the imagination of crisis, termed Inhibitory Ethical Risk Perception (PI). This aligns with Regulatory Focus Theory^20^and the challenge hindrance stressor framework^21^..

Inhibitory Ethical Risk Perception (PI): Refers to educators’ hindrance assessment of ethical risks such as disrupted teaching fairness, weakened student abilities, and data privacy breaches caused by AI. Promotional Professional Risk Perception (PP): Drawing on the Challenge Stressor framework^21^, it extends beyond threat to encompass challenge assessment arising from the concern about outdated teaching methods or a decline in professional competitiveness, mobilizing resources for adaptation. The two are conceptually distinct: PI assesses ethical risk directly, while PP is a challenge oriented evaluation geared toward opportunities.

Faced with dual ethical risks, educators’ coping behaviors also exhibit strategic differentiation^7^. Drawing on Witte’s^22^Extended Parallel Process Model (EPPM), widely applied in technological threat research^23^, we propose two ethical coping strategies aligned with AIED ethical frameworks^24^: Defensive Ethical Coping (ED): Protective, avoidant, and restrictive strategies to reduce exposure to ethical risks, reflecting technology threat avoidance patterns^25^. Constructive Ethical Coping (EC): Proactive approaches aimed at enhancement and integration, which address ethical risks in a highly effective manner, such as redesigning assignments to be AI resistant.

For detailed operationalization and theoretical foundations of these constructs, please refer to Supplementary Table S1.

### AI literacy and the literacy sedative effect

This study focuses on AI Literacy (AS) as a key personal resource, defined as a multidimensional ability^26^. Recent literature has emphasized its importance in proficiently addressing AI challenges^27^. Beyond the classic TPACK framework^8^,AI Literacy combines objective technical knowledge with prudent ethical judgment^9^. The main mechanism for this cautious evaluation lies in the activation of reflective agency^28^. In theory, this process suppresses the automatic dominance of System 1 (emotional heuristics) while actively mobilizing System 2 (evidence based analytical reasoning)^29,30^. The indirect research results also support this point, indicating that high level digital competence helps reduce individuals’ dependence on emotional heuristics^31^. This cognitive transformation helps buffer emotional amplification, thus forming the theoretical basis for the Literacy Sedative Effect. Therefore, investing cognitive resources in Defensive Coping is not a manifestation of insufficient ability, but a rational manifestation of high ability and sense of responsibility in complex situations.

### Contextual boundaries and the reverse Matthew Effect

In addition to AI Literacy, organizational context is also a key boundary condition that challenges the classical Matthew Effect^10^. The academic community’s attention to the digital divide has evolved from the traditional gap in access and skills^11^to the third level digital divide characterized by differences in technology usage outcomes^12^. This perspective emphasizes that the key to determining the final outcome is not simply resource ownership, but rather technological agency (the ability to transform technological potential into actual value). Therefore, this study proposes the Reverse Matthew Effect hypothesis, which suggests that schools with scarce resources may exhibit stronger technological agency. The organizational logic of this phenomenon is rooted in institutional logic and Conservation of Resources (COR) theory^32,33^: survival pressure prompts scarce schools to form specific breakthrough logic, viewing AI as a strategic mechanism for achieving accelerated organizational advancement^34^. On a psychological level, they may also perceive higher task value^35^. However, the activation of this resource breakthrough mechanism may have identity boundaries. According to the theory of organizational socialization^36^, in-service teachers have entered the encounter stage, and they are directly immersed in the institutional reality of the school, thus experiencing the survival pressure brought by resource scarcity firsthand. In contrast, pre-service teachers are still in the anticipatory socialization stage^37^, and their perception of occupational risks is based more on imagination of the future rather than immediate institutional constraints. Consequently, this theoretical distinction suggests that the Reverse Matthew Effect, as a survival response to objective scarcity, is likely to be more salient in in-service teachers than in pre-service teachers. In addition, to ensure the robustness of the model, this study also incorporated the complex effects of demographic variables, such as the nonlinear effects of age (life course perspective^38^ and gender differences (social role theory^39^.

**Fig. 1:**
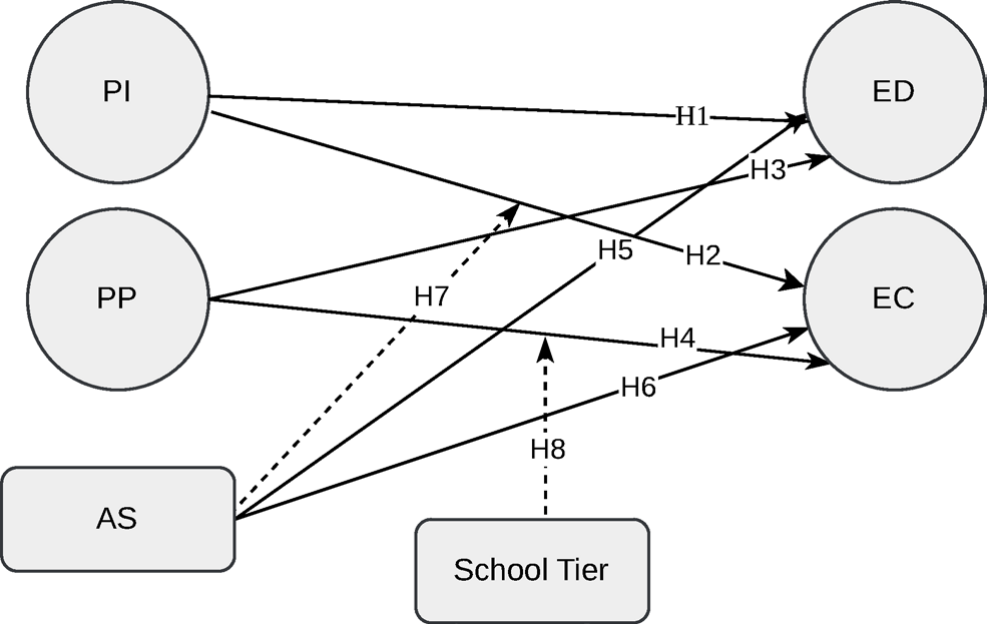
The hypothesized theoretical model of educators’ ethical coping. Arrows represent hypothesized paths (H1–H6) and moderating effects (H7–H8) among risk perceptions (PI, PP), AI literacy (AS), and coping strategies (ED, EC). H1 & H2: Inhibitory Ethical Risk perception (PI) positively predicts Defensive Coping (ED) (H1) and Constructive Coping (EC) (H2). H3 & H4: The perception of Promotional Professional Risk (PP) is a positive predictor of Defensive Coping (ED) (H3) and Constructive Coping (EC) (H4). H5 & H6: AI Literacy (AS) is a positive predictor of Defensive Coping (ED) (H5) and Constructive Coping (EC) (H6). H7 (Literacy Sedative Effect): The positive association between Inhibitory Ethical Risk perception (PI) and Constructive Coping (EC) is expected to be attenuated by AI Literacy (AS). H8 (Reverse Matthew Effect): The positive association between Promotional Professional Risk Perception (PP) and Constructive Coping (EC) is expected to be stronger in lower tier schools.

In summary, this study constructs a comprehensive framework for analyzing educators’ ethical coping in the AI era. This framework not only defines the duality of risk perception and the differentiation of coping strategies, but also proposes two prospective moderating mechanisms: the Literacy Sedative Effect at the individual level and the Reverse Matthew Effect at the contextual level. Based on this, this study proposes the following core hypotheses (Fig. 1 visually illustrates the theoretical model):

## Methods

### Participants and procedure

This study collected data from 502 Chinese in-service and pre-service educators through a questionnaire survey. To ensure sample heterogeneity, participants were drawn from primary and secondary schools in mainland China (central, eastern, and southern regions), and a comprehensive university in southwestern China (see Table 1 for detailed demographic characteristics). For pre-service teachers, the school tier was recorded based on their internship schools. Specifically, school tiers are classified according to China’s administrative system and educational resource allocation. Tier 1 refers to key public schools at the provincial and municipal levels with abundant resources in first tier cities such as Beijing and Shanghai. Tier 2 represents schools in provincial capitals, while Tier 3 comprises schools in prefecture level cities. Tier 4 includes county level schools or private institutions with relatively limited public funding. To preclude potential bias in sample distribution, we specifically examined the composition of Tier 4 (resource constrained schools). The data shows that 88.1% of participants in this tier are in-service teachers, while only 11.9% are pre-service teachers. This high proportion of in-service teachers ensures that the effects observed at this level mainly reflect the authentic logic of professional survival, rather than the temporary characteristics of pre-service teachers. Although the sample age distribution shows a trend towards younger age groups (approximately 57.4% of respondents are under 30 years old), this is highly consistent with the demographic characteristics of early adopters of generative AI technology. This sample structure helps to accurately capture the cognitive landscape of digital natives, providing valuable forward looking perspectives for predicting the evolution trend of future educational technology integration.

**Table 1:** Participant demographics (*N* = 502).

Variable	Category	*n*	%
**Identity**	K-12 Teacher (≤ 3 yrs)	87	17.33
	K-12 Teacher (4–10 yrs)	75	14.94
	K-12 Teacher (≥ 11 yrs)	91	18.13
	Higher-Ed Teacher	24	4.78
	Pre-service Teacher (Undergraduate)	162	32.27
	Pre-service Teacher (Graduate)	35	6.97
	Educational Support Staff	28	5.58
**Gender**	Female	275	54.78
	Male	227	45.22
**Age Group**	< 30	288	57.37
	30–39	144	28.69
	40–49	52	10.36
	≥ 50	18	3.59
**School Tier**	Tier 1 (Top)	103	20.52
	Tier 2 (Mid)	88	17.53
	Tier 3 (Regular)	269	53.59
	Tier 4 (County Private)	42	8.37

*Note*. This table presents the frequency (*n*) and percentage (%) distribution of the sample across key demographic variables.

This study strictly followed ethical standards (approved by the IRB and with informed consent obtained from all participants). After optimizing the questionnaire through pilot testing, a three week data collection was conducted using the Wenjuanxing platform in August 2025. To ensure sample heterogeneity, a combination of stratified convenience sampling and snowball sampling was used in the study. Questionnaires were distributed to school leaders and key teachers from different institutional backgrounds (covering developed coastal cities to resource constrained inland counties) through professional teacher networks on WeChat, and were further forwarded to colleagues. All participation followed the principles of voluntariness and anonymity to reduce social desirability bias.

To ensure the rigor of the data, we conducted a strict two stage data cleaning procedure on the 533 original questionnaires collected. Phase 1: Eliminate invalid answers (*N* = 533 → 508). In accordance with methodological recommendations, we removed any samples with response times below 60 s. This limit was derived from 80% of the shortest pilot completion time to verify sufficient cognitive engagement. Phase 2: Eliminate logically contradictory samples (*N* = 508 → 502). To ensure data quality, we used auxiliary validation items within the questionnaire (such as detecting conflicting responses between overall attitudes and specific behaviors) to identify and eliminate six samples with logical inconsistencies. After this screening, 502 valid samples were ultimately retained.

### Measures

All core constructs were assessed utilizing a 7 point Likert scale, with scales either adapted from established literature or developed on robust theoretical foundations, unless specified otherwise. Given that the original scales were primarily developed in the English context, this study followed a standardized scale revision procedure. Firstly, two bilingual researchers independently translated the items into Chinese, and an independent translator performed an English back translation to ensure semantic equivalence. Subsequently, a linguist and an educational technology expert reviewed the revised items to ensure their contextual validity in the context of generative AI education in China. Before finalizing the measurement tool, this study conducted a pilot test (*N* = 20) to verify the clarity of all items and their comprehensibility for respondents. The following are the number of items, reliability (Cronbach’s *α*), and sources for the respective constructs:

Inhibitory Ethical Risk Perception (PI): 4 items, *α* = 0.800, adapted from Schepman & Rodway^40^and Wang & Wang^41^..

Promotional Professional Risk Perception (PP): 4 items, *α* = 0.777, adapted from Wang & Wang^41^ and developed for this study.

Defensive Coping (ED): Consisting of 3 items, *α* = 0.704, adapted from Wang et al.^42^ and developed for this study.

Constructive Coping (EC): 4 items, *α* = 0.885, adapted from Chiu et al.^43^, Wang et al.^42^ and developed for this study.

AI Literacy (AS): 8 multiple choice questions assessing objective knowledge and skills, from Jin et al.^44^ (see Supplementary Table S2 for full items and Table S3 for item statistics). It was evaluated using an objective knowledge test (score range: 0–8). The final score for each participant was the total number of questions they answered correctly. Based on the analysis of the final sample (*N* = 502), the difficulty (pass rate) of the questions ranged from 52.99% to 74.10% (*M* = 4.93, *SD*= 1.98), indicating that the test difficulty was moderate and effectively avoided floor or ceiling effects. The KR-20 reliability coefficient was 0.605. Although this is slightly lower than the commonly used threshold for traditional homogeneous scales, it exceeds the 0.60 benchmark recommended for exploratory studies and newly developed tools^45^. In addition, considering that the test is a broad spectrum formative indicator covering highly heterogeneous domains (such as technical principles and ethical norms) rather than a single latent variable, a lower internal consistency is theoretically expected and fully acceptable in psychometrics^46^. In PLS-SEM analysis, AS is modeled as a single indicator total score variable representing the comprehensive knowledge level of educators. More importantly, from a psychometric perspective, moderate reliability often leads to an attenuation of the observed effect size. Therefore, the significant moderating effect captured in the model should be considered as a robust conservative estimate, which suggests that the actual Literacy Sedative Effect may be stronger than the observed value.

The complete items and sources for all core scales in this study are detailed in the Supplementary Tables S2 and S3.

### Data analysis

We used SmartPLS (v. 4.1.1.6)^47^ for PLS-SEM analysis, and chose this method because it outperforms covariance based SEM in three key aspects: its predictive ability (*R*², *Q*²)^48^, robustness in estimating complex interaction effects^49^, and flexibility in handling formative measures such as AI Literacy. This study performed a power analysis with G*Power 3.1 to confirm the statistical power of the sample size. The minimum sample size needed for the most complicated model with predictor variables and interaction terms was 109. This was based on the criteria of moderate effect size *f*² = 0.15, significance level alpha = 0.05, and power level 0.80. The final sample size of 502 participants in this study far exceeded this threshold, with a statistical power greater than 0.99. This shows that the current sample size is adequate to find significant effects, which means that the PLS-SEM analysis is strong.

The analysis process strictly followed the two step approach. In the first step, we evaluated the measurement model by assessing internal consistency using Cronbach’s alpha and composite reliability (CR) (or KR-20 for the objective knowledge index), convergent validity using Average Variance Extracted (AVE), and discriminant validity using the stringent Heterotrait-Monotrait Ratio (HTMT) criterion^50^ to compensate for the limitations of the traditional Fornell-Larcker criterion. Additionally, to rigorously assess common method bias (CMB), we employed a dual approach combining Harman’s single factor test and Full Collinearity Assessment (VIF). In the second step, the structural model was estimated using the path weighting scheme with a maximum of 3,000 iterations and a stop criterion of 10^− 7^. Significance testing was performed using a bootstrapping procedure with 5,000 subsamples and bias-corrected and accelerated (BCa) confidence intervals to ensure the robustness of the path coefficients. Additionally, we reported *f*² (effect size) to evaluate the substantial impact of variables and calculated *Q*^2^_Predict_ using the PLSpredict procedure to validate the out of sample predictive relevance of the model^51^. For the interaction effects, in addition to testing the significance of the interaction term, this article also conducted a Johnson-Neyman (J-N) analysis using a Python (v3.10) script to determine the significance boundaries. In terms of technical details, OLS regression and *t*-distribution estimation were performed using scikit-learn (v1.3.0) and scipy (v1.11.0); numpy (v1.26.0) was used to solve quadratic equations and lock the critical points; *α* = 0.05 was set to calculate the 95% confidence band and it was visualized using matplotlib (v3.8.0). Finally, robustness tests were conducted by controlling for age and gender to eliminate confounding effects.

## Results

### Descriptive and preliminary analyses

The background information of the respondents is shown in Table 1. The mean, standard deviation, and correlation coefficient matrix of each core latent variable are detailed in Table 2. The results showed that educators exhibited a high tendency towards action in both Constructive Coping (*M* = 5.50, *SD* = 1.00) and Defensive Coping (*M* = 5.31, *SD* = 0.97). Interestingly, educators reported identical levels of Inhibitory Ethical Risk (PI) and Promotional Professional Risk (PP) (*M* = 5.06). While this suggests a balanced perception of AI’s challenges and opportunities, the difference in standard deviations (1.07 vs. 1.04) indicates distinct patterns of variability in how these risks are experienced. Educators reported a moderate level of objective AI Literacy (*M* = 4.93, *SD* = 1.98). The correlation analysis showed that there is a significant correlation between most core variables. Specifically, AI Literacy (AS) showed significant positive correlations with Constructive Coping and Defensive Coping. There is a strong positive correlation between Defensive and Constructive Coping (see Table 2).

**Table 2:** Descriptive statistics and correlations.

Construct	Mean	SD	1	2	3	4	5
1. Inhibitory Ethical Risk (PI)	5.06	1.07	-				
2. Promotional Professional Risk (PP)	5.06	1.04	0.65**	-			
3. Defensive Coping (ED)	5.31	0.97	0.50**	0.56**	-		
4. Constructive Coping (EC)	5.50	1.00	0.40**	0.50**	0.70**	-	
5. AI Literacy (AS)	4.93	1.98	0.10*	0.11*	0.17**	0.26**	-

*Note*. *N* = 502. PI = Inhibitory Ethical Risk; PP = Promotional Professional Risk; ED = Defensive Coping; EC = Constructive Coping; AS = AI Literacy. Scales: PI, PP, ED, EC (1–7); AS (0–8, total correct). It is coincidental that PI and PP share the same rounded mean (5.06). They are distinct constructs with different standard deviations (1.07 vs. 1.04) and discriminant validity. *p < .05, **p < .01.

In terms of demographic variables, although preliminary *t*-tests showed significant gender differences (female educators had slightly stronger Constructive Coping) and age differences (coping levels peaked at 30–49 years old), in order to ensure the robustness of the core theoretical relationship and eliminate interference, subsequent structural equation model has included these factors as control variables.

### Evaluation of the measurement model

This study used SmartPLS 4^47^ for partial least squares structural equation modeling (PLS-SEM) analysis. Firstly, we evaluated the reliability and validity of the measurement model (see Table 3). The Cronbach’s alpha and composite reliability (CR) of all latent variables exceeded or met the recommended threshold of 0.70^48^, indicating satisfactory internal consistency. AS was modeled as a single indicator formative index; detailed statistics (KR-20 = 0.605) are in Supplementary Table S3. In terms of convergent validity, all items exhibited factor loadings above the 0.70 threshold, and the average variance extracted (AVE) of each variable was greater than 0.50, confirming robust convergent validity.

Discriminant validity was assessed using the Heterotrait-Monotrait (HTMT) ratio of correlations. It should be acknowledged that the HTMT value between ED and EC is 0.886. While this exceeds the conservative threshold of 0.85, placing their discriminant validity at a borderline level, it remains below the recommended threshold of 0.90 for conceptually similar constructs^50^. This high correlation reflects the parallel nature of coping responses in our theoretical framework. More importantly, based on 5,000 bootstrap resamples, the upper bound of the 95% confidence interval for HTMT is 0.948, strictly excluding 1.0. According to the inference criterion by Henseler et al.^50^, this is the ultimate criterion for determining discriminant validity. This statistical result, combined with the heterogeneity exhibited by the two variables in the predictive model, confirms that they are independent behavioral dimensions rather than different facets of the same dimension. The upper bound of the CI for all other constructs was well below 1.0, demonstrating satisfactory discriminant validity (Table 4).

**Table 3:** Measurement model evaluation.

Construct	Items	Loading	Cronbach’s Alpha	CR	AVE
Inhibitory Ethical Risk (PI)	PI1	0.783	0.800	0.869	0.623
	PI2	0.785			
	PI3	0.830			
	PI4	0.757			
Promotional Professional Risk (PP)	PP1	0.705	0.777	0.853	0.593
	PP2	0.810			
	PP3	0.722			
	PP4	0.834			
Defensive Coping (ED)	ED1	0.803	0.704	0.835	0.628
	ED2	0.748			
	ED3	0.825			
Constructive Coping (EC)	EC1	0.846	0.885	0.921	0.744
	EC2	0.866			
	EC3	0.869			
	EC4	0.868			

*Note*. CR = Composite Reliability; AVE = Average Variance Extracted. Cronbach’s alpha is not reported for AS (objective sum score) as traditional internal consistency metrics are not applicable.

**Table 4:** Discriminant validity (HTMT and 95% Bias-corrected confidence intervals).

Relationship	HTMT Ratio	95% CI (Bias-Corrected)	Discriminant Validity
ED ↔ EC	0.886	[0.811, 0.948]	Yes
PI ↔ AS	0.105	[0.050, 0.194]	Yes
PI ↔ EC	0.472	[0.354, 0.573]	Yes
PI ↔ ED	0.662	[0.558, 0.760]	Yes
PP ↔ AS	0.112	[0.047, 0.169]	Yes
PP ↔ EC	0.562	[0.457, 0.658]	Yes
PP ↔ ED	0.722	[0.617, 0.818]	Yes
PP ↔ PI	0.844	[0.764, 0.912]	Yes

Note: *N* = 502 (bootstrap samples = 5,000). HTMT = Heterotrait-Monotrait Ratio; CI = Confidence Interval. Abbreviations: ED = Defensive Coping; EC = Constructive Coping; PI = Inhibitory Ethical Risk Perception; PP = Promotional Professional Risk Perception; AS = AI Literacy. Discriminant validity is established if the HTMT confidence interval strictly excludes 1.0.

### Evaluation of the structural model

**Fig. 2:**
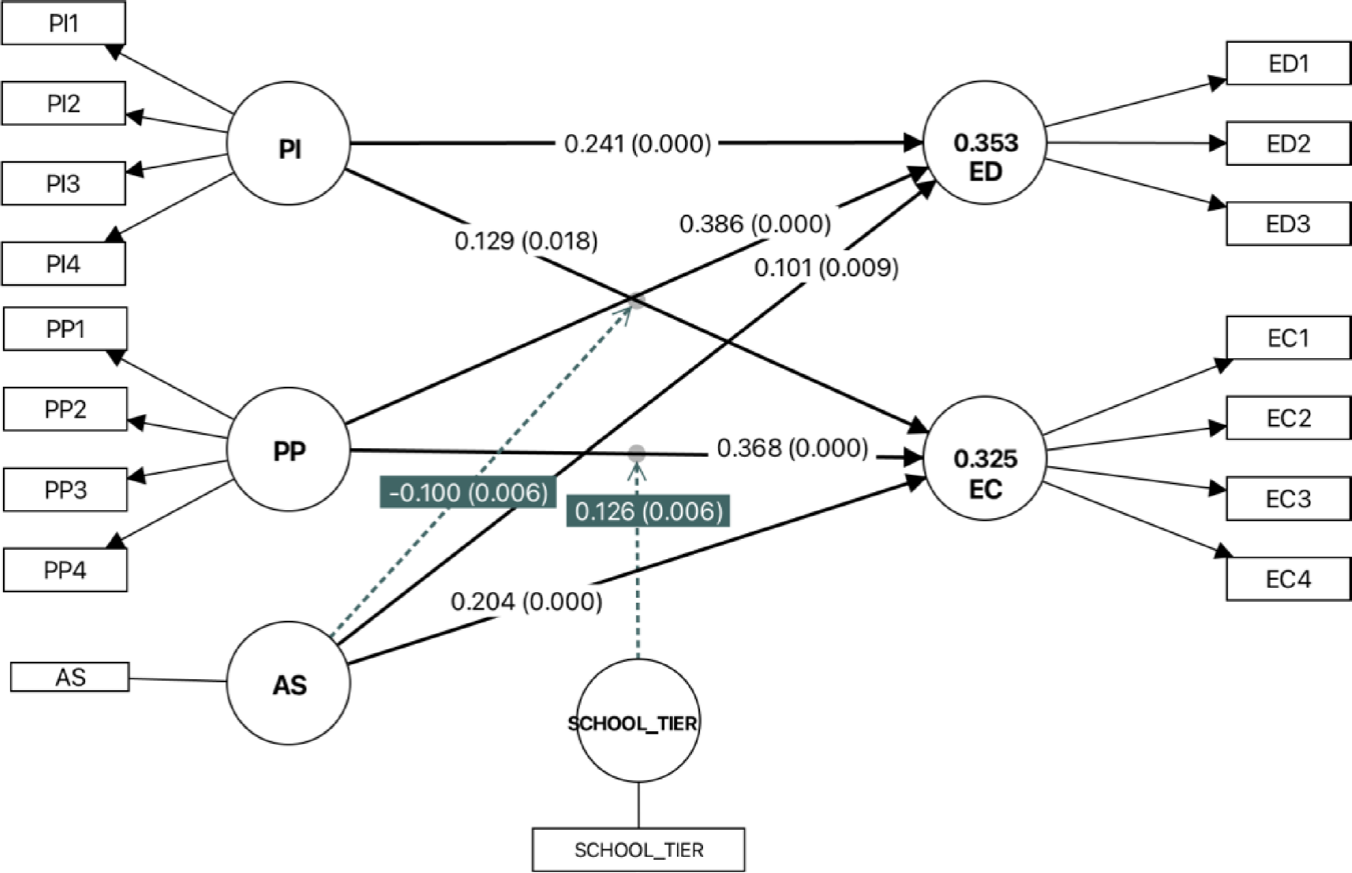
PLS-SEM structural model results (*N* = 502).

To ensure the robustness of the data, this study employed a dual strategy to rigorously evaluate common method bias (CMB). Harman’s single factor test showed that the first unrotated factor explained 42.73% of the variance, below the critical value of 50%^52^. In addition, collinearity diagnostics showed that the variance inflation factor (VIF) values of all predictor constructs in the structural model ranged from 1.01 to 1.79, all far below the recommended threshold of 3.3^53^.These results consistently indicate that common method bias is not a pervasive issue in this study, confirming the data quality for structural model assessment. Model fit was assessed using the standardized root mean square residual (SRMR). The SRMR value for the saturated model was 0.070, which is lower than the recommended threshold of 0.08, indicating that the model fits well^48^. This model has moderate explanatory power for the variance of Defensive Coping (*R*² = 0.353) and Constructive Coping (*R²* = 0.325). More importantly, the out of sample predictive ability evaluated by the PLSpredict program^51^ showed that the *Q*² values for Defensive Coping (*Q*^2^_Predict_ = 0.337) and Constructive Coping (*Q*^2^_Predict_ 0.304) were significantly greater than 0, confirming the high predictive relevance of the model. Given the large sample size (*N* = 502, statistical power > 0.99), this study strictly distinguished statistical significance from practical significance by reporting effect sizes (*f*^2^). Although all paths reached statistical significance (*p* <.05), compared to the weaker effect sizes of PI and AS (*f*^2^: 0.014–0.061), Promotional Professional Risk (PP) showed a more substantial practical impact on the two coping strategies (*f*^2^: 0.112–0.131). This indicates that PP is the core practical driving factor in this model (Fig. 2).

The path coefficients and hypothesis testing results are detailed in Table 5. The results showed that all eight core structural paths reached statistical significance, strongly supporting the theoretical framework. Specifically:

The associations of risk (H1–H4): Both Inhibitory Ethical Risk (PI) and Promotional Professional Risk (PP) were significantly and positively associated with Defensive and Constructive Coping strategies. It is worth noting that the positive association of Promotional Professional Risk is stronger than that of Ethical Risk in both types of coping.

The role of AI Literacy (H5–H6): AI Literacy (AS) is also a significant positive predictor of Defensive and Constructive Coping strategies.

Interaction effects (H7–H8): The Literacy Sedative Effect and the Reverse Matthew Effect were statistically significant (see moderation analysis below for details).

**Table 5:** Structural model results and hypothesis testing.

Hypothesis	Path	β	SD	*t*-value	*p*-value	95% CI (BC)	Result
**Structural Paths**							
H1	PI → ED	0.241	0.055	4.410	< 0.001	[0.134, 0.345]	Supported
H2	PI → EC	0.129	0.054	2.373	0.018	[0.027, 0.237]	Supported
H3	PP → ED	0.386	0.056	6.893	< 0.001	[0.277, 0.498]	Supported
H4	PP → EC	0.368	0.053	6.992	< 0.001	[0.262, 0.468]	Supported
H5	AS → ED	0.101	0.039	2.626	0.009	[0.026, 0.174]	Supported
H6	AS → EC	0.204	0.036	5.735	< 0.001	[0.134, 0.275]	Supported
**Interaction Terms**							
H7	AS × PI → EC	−0.100	0.036	2.750	0.006	[−0.169,−0.025]	Supported
H8	Tier × PP → EC	0.126	0.045	2.777	0.006	[0.032, 0.209]	Supported

*Note*. *β* = Standardized path coefficient; *SD* = Standard Deviation; CI = Bias-Corrected Confidence Interval (based on 5,000 bootstrap samples). Abbreviations: PI = Inhibitory Ethical Risk Perception; PP = Promotional Professional Risk Perception; AS = AI Literacy. Effect sizes (*f*^2^) for significant paths ranged from 0.014 to 0.131. All hypothesized paths were statistically significant.

### Moderation analysis

The moderation analysis results substantiate the two core interaction mechanisms proposed in this study.

**Fig. 3:**
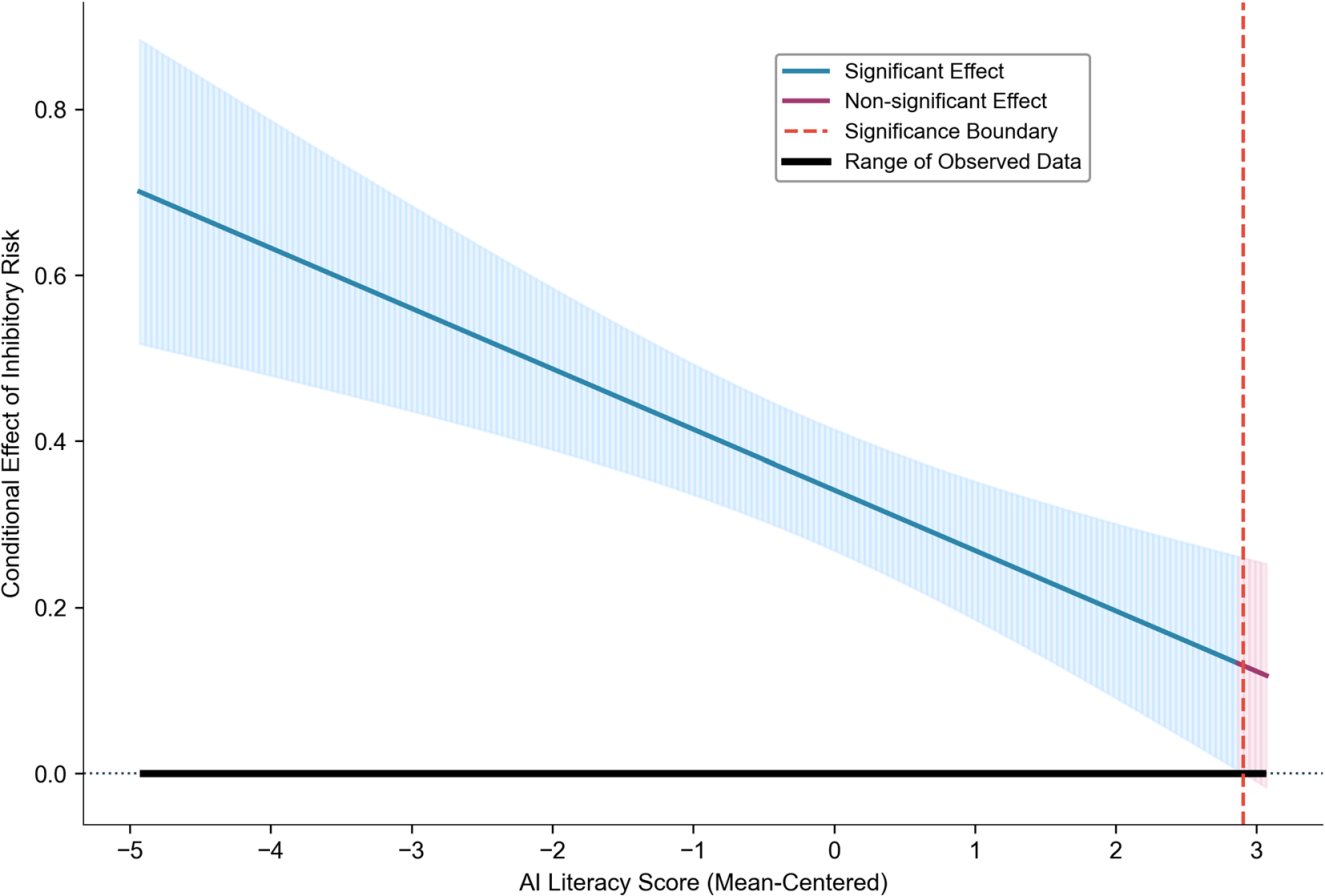
Johnson-Neyman plot for the interaction between AI literacy and inhibitory ethical risk. The shaded area represents the 95% confidence intervals. The vertical dashed line indicates the significance boundary (mean-centered score = 2.91), beyond which the positive effect of ethical risk on constructive coping becomes statistically non-significant.

Firstly, the Literacy Sedative Effect (H7): AI Literacy plays a significant negative moderating role between Inhibitory Ethical Risk and Constructive Coping (see Table 5). Considering the reliability of the AI literacy measurement tool (KR-20 = 0.605), this study applied a correction for attenuation. The estimated theoretical path coefficient was approximately − 0.152, indicating that the observed effect size was underestimated by 34.3% due to measurement error. Figure 3 visualizes the interaction effect based on mean centered data using the Johnson-Neyman (J-N) technique. The chart shows a clear fan shaped pattern: for educators with low AI Literacy, Inhibitory Ethical Risk is significantly and positively associated with Constructive Coping; however, with the improvement of AI Literacy, this positive association is gradually diminished. J-N analysis accurately identified the significance boundary: when the AI Literacy score of educators is higher than the significance threshold of 2.91 (Mean Centered Score > 2.91), the 95% confidence interval contains zero, and the positive association of Inhibitory Ethical Risk is no longer statistically significant.

**Fig. 4:**
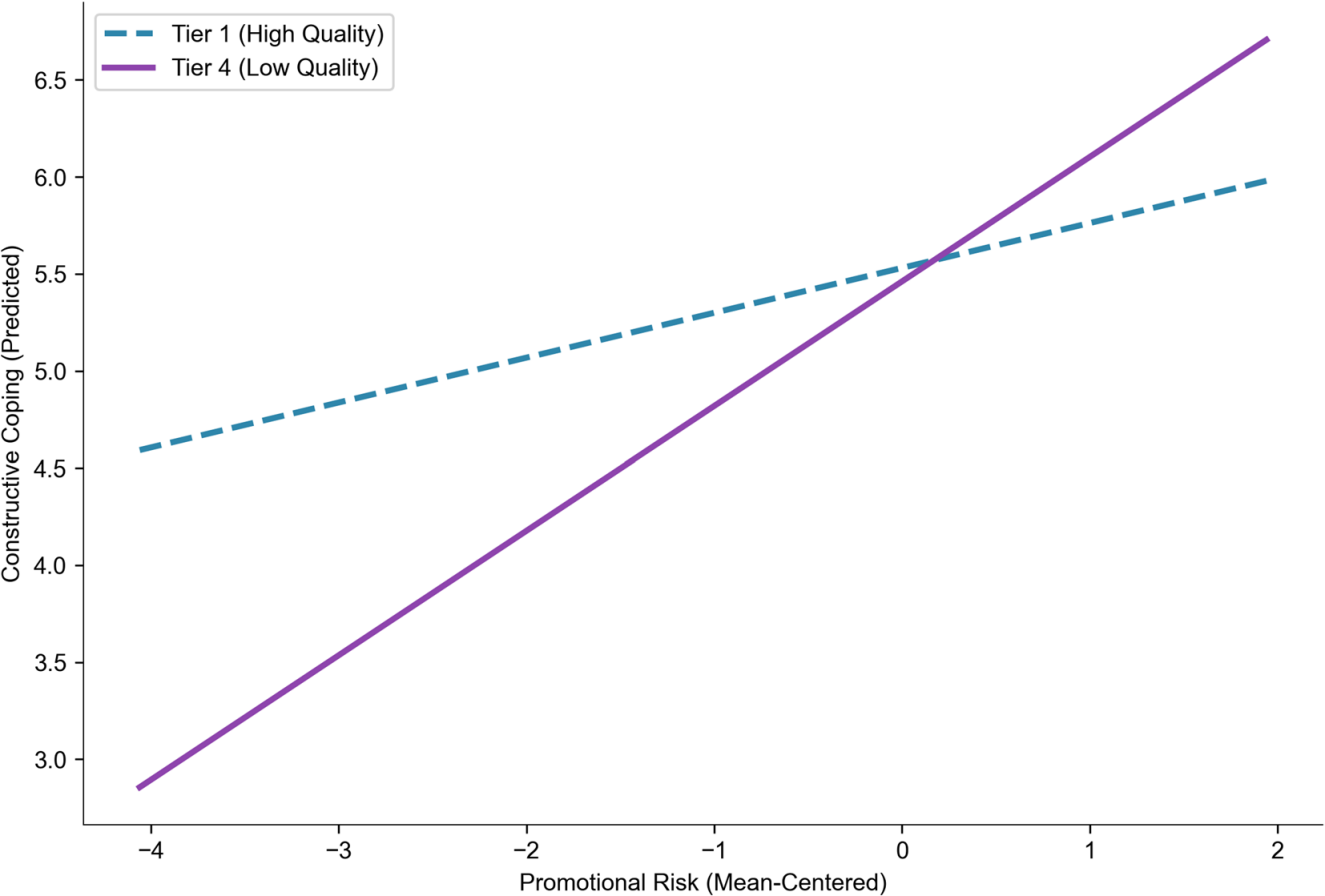
Simple slope analysis of the moderating effect of school tier. The steeper PP–EC regression slope for Tier 4 versus Tier 1 schools indicates stronger technological agency under resource scarcity.

Secondly, The Reverse Matthew Effect (H8): The school tier plays a significant positive moderating role between Promotional Professional Risk and Constructive Coping (see Table 5). Figure 4 provides strong visual evidence through simple slope analysis. As shown in the figure, compared to the relatively gentle slope (Slope ≈ 0.231) of high quality schools in first tier cities (Tier 1, blue dashed line), educators in non-first tier or weak schools (Tier 4, purple solid line) have a steeper regression slope (Slope ≈ 0.642) in their Constructive Coping when perceiving Promotional Professional Risks. Specifically, in resource constrained environments, the positive association of Promotional Professional Risk is 2.78 times stronger than in resource enriched environments. In addition, to ensure that this effect is not an artifact caused by continuous variable modeling assumptions or uneven sample distribution across school tiers, an additional sensitivity analysis was conducted. We temporarily reorganized the sample into two macro groups with balanced group sizes, specifically the resource advantaged group (Tier 1 & 2, *n* = 191) and the resource constrained group (Tier 3 & 4, *n* = 311). Multi group interaction analysis robustly confirmed this disparity. Compared to the resource advantaged group (*β* = 0.276, *p* <.001), the resource constrained group exhibited a steeper positive slope (*β* = 0.540, *p*<.001; interaction *p* =.001). Moreover, to eliminate the confounding effects of demographic characteristics, this study conducted robustness tests. After controlling for the variables of age and gender, the interaction effect remained significant. Finally, in response to potential concerns regarding the inclusion of pre-service teachers (39.2%) in the sample, we conducted a sensitivity analysis. Rather than reducing the sample size, we introduced a dummy variable, Identity (0 = In-service Teacher, 1 = Pre-service Teacher) as a control covariate in the interaction models. The results demonstrated that even after controlling for identity, the core interaction effects remained robust: the moderating effect of AI Literacy on Inhibitory Ethical Risk (Literacy Sedative Effect) remained significant (*β* = −0.076, *p* <.01), and the moderating effect of School Tier on Promotional Professional Risk (Reverse Matthew Effect) remained highly significant (*β* = 0.109, *p* =.004). To further deepen the validation, we conducted a multi-group analysis. The results showed that the Literacy Sedative Effect remained significant in both groups (in-service: *β* = −0.176; pre-service: *β* = −0.137), suggesting its robustness across different professional stages as a cognitive mechanism. In contrast, the Reverse Matthew Effect exhibits distinct group specificity: it is only significant among in-service teachers (*β* = 0.164, *p* =.001), and not significant at all among pre-service teachers (*β* = 0.011, *p* =.867).

## Discussion

The core contribution of this study is to systematically reveal the complex ethical coping mechanisms of educators in the AI era. By constructing a dual-dimensional risk-coping framework, our findings collectively identify a behavioral pattern of cautious innovation, wherein individuals navigate these technological disruptions by simultaneously embracing change while establishing safeguards. Moving beyond surface level applied research, this study uncovers deep cognitive mechanisms through two key findings: the Literacy Sedative Effect and the context driven Reverse Matthew Effect, which jointly respond to the urgent agenda of AI ethics.

The primary theoretical contribution of this study is the proposal of a dual-dimensional risk-coping framework. The framework surpasses the limitations of traditional technology acceptance models, such as TAM. It responds to the academic call for an in depth exploration of underlying ethical and cognitive mechanisms^16,17^. These mechanisms are recognized as being central to the current discussion. To accomplish this, the framework operationalizes a macro level theory at the individual psychological level. It specifically translates the concept of sociotechnical imaginaries^4^into Promotional Professional Risk (PP) and Inhibitory Ethical Risk (PI)^19^..

Our framework reveals that true professional competence in the high risk context of AI ethics is a manifestation of both offensive and defensive wisdom. Although all theorized paths reached statistical significance, there are differences in the magnitude of their effects, with professional risk perception showing higher practical significance in driving constructive adaptation. The finding that AI Literacy predicts both defensive prudence and constructive innovation challenges the simplistic assumption that ability unidirectionally predicts technological adoption. Crucially, the substantial positive association between Defensive and Constructive Coping provides profound insights into the paradox of parallel activation. Unlike traditional Stress Theory^7^ that views fight or flight as competing mechanisms, our research reveals a symbiotic relationship in the context of rapid AI integration: educators are not choosing between defense and construction, but are compelled to pursue a path of prudent innovation.

The analysis supports the Literacy Sedative Effect, indicating that teachers’ professional abilities are surpassing the classical TPACK framework^8^and evolving towards higher order AI Literacy centered on prudent judgment^9^. Empirical evidence indicates that objective AI Literacy attenuates the positive relationship between Inhibitory Ethical Risk and Constructive Coping via cognitive decoupling mechanisms^54^. Specifically, drawing on Dual Process Theory^30^, high literacy educators are able to suppress System 1 (fast thinking) that relies on emotional heuristics^29^and instead engage System 2 (slow thinking) characterized by thoughtful and rational analysis. Therefore, their coping strategies are detached from immediate emotional reactions. Thus, the primary goal of enhancing AI Literacy is not to foster uncritical technological optimism. Rather, its purpose is to develop reflective practitioners who can thoughtfully assess and adeptly manage risks^28^..

The findings regarding the Reverse Matthew Effect provide a fresh perspective on the traditional belief that resource advantage equals innovation advantage^10^,and provides strong empirical support for the third level digital divide theory, where outcomes are driven by agency rather than just resources^12^. However, given that this study uses school tier as an administrative proxy variable, the following discussion on resource scarcity, institutional pressure, and technological agency is intended as a post-hoc theoretical interpretation based on empirical results, rather than the causal verification of direct measurement mechanisms. One possible explanation lies in the conflict of institutional logics^32^: the conservative performance logic of resource rich schools is opposed to the breakthrough logic formed by resource poor schools under pressure^33^,which corresponds with significantly stronger technological agency, motivating them to view AI as a catalyst for accelerated development^34^. In addition, this breakthrough logic not only stems from psychological motivation, but may also be closely related to the coercive nature of organizational management. In order to compensate for resource disadvantages, Tier 4 schools often implement more aggressive top down directives, viewing AI as a necessary option to improve efficiency, thereby structurally strengthening teachers’ constructive coping; in contrast, due to the need to maintain reputation, Tier 1 schools may face stricter institutional regulations and risk avoidance policies, which in turn buffer the aggressiveness of technology applications. This conclusion has been further validated by multi-group analysis where the Reverse Matthew Effect disappears in the pre-service teacher group lacking professional pressure. This null result conversely suggests that technological agency is not an inherent characteristic of the younger generation but rather a professional survival logic inspired by resource constrained environments. This finding posits technological agency, not merely resources, as the core driver of technological integration outcomes, thus offering a new theoretical lens to examine educational equity in the AI era.

The findings suggest three important implications for practice. First, policymakers should pursue a dual track strategy: mitigate Inhibitory Ethical Risk perception with clear guidelines while channeling Promotional Professional Risk Perception into innovation via dedicated platforms. Second, informed by the Literacy Sedative Effect, teacher training needs to go from teaching AI skills in isolation to teaching AI Literacy in a systematic way that focuses on core skills like threat assessment, ethical judgment, and redesigning lessons. Finally, the Reverse Matthew Effect implies administrators should not equate resources with innovation, and instead tilt policy support toward highly motivated, lower tier schools to foster grassroots innovation.

This study also has several limitations that need to be addressed in future research: (1) This cross-sectional study cannot establish strict causality. To empirically demonstrate directional superiority, we specified a reverse path model (coping → risk perception) for a formal competitive model test. The results showed a significant deterioration in global fit indices, with the standardized root mean square residual (SRMR) of the saturated model increasing from 0.070 in the original forward model to 0.076 in the reverse model, and the estimated model fit also deteriorating from 0.102 to 0.113. These fit indices provide statistical evidence that the theorized causal direction (perception → coping) more accurately reflects the observed data structure than reverse causality. We acknowledge that there may be dynamic reciprocity between perception and behavior from a long term perspective. Subsequent research should adopt longitudinal designs to capture this feedback loop; for example, the recent study by Liu et al.^55^ provided a robust methodological framework for examining such dynamic causal mechanisms. In addition, the reliance on self-report data can be compensated for in the future through experimental methods or classroom observation studies. (2) The objective AI literacy test used in this study showed moderate reliability. Although this value is acceptable for broad spectrum formative indicators and has been statistically addressed through attenuation correction, this measurement noise inherently limits the precision of effect size estimation. Future research should focus on developing and validating homogeneous subscales with higher internal consistency. (3) The single level analysis of school tiers is exploratory. Due to the reliance on school tier as an administrative proxy variable, the empirical basis of the Reverse Matthew Effect is somewhat limited. Because we did not directly quantify the underlying mechanisms proposed (i.e., objective resource scarcity, specific institutional pressures, and subjective technological agency), the relevant discourse is actually a post-hoc theoretical interpretation. Future research should adopt multilevel models and directly measure organizational climate variables (such as the intensity of management policies, e.g., mandatory AI usage requirements) to elucidate the interaction between individual agency and institutional constraints. (4) The study’s conclusions were drawn from a Chinese context, and the sample age distribution is biased towards young educators. Future research needs to further validate the cross-cultural generalizability of these findings and ensure broader representation among senior teachers.

## Conclusion

The core conclusion of this study is that educators’ effective coping with ethical challenges in AI is a dynamic balance between prudent defense and proactive construction. This conclusion deepens existing theory on two fronts: First, the identification of the Literacy Sedative Effect reveals that teacher professional competence is surpassing the classic TPACK framework and evolving into a higher order AI Literacy centered on prudent judgment. Second, by substantiating the Reverse Matthew Effect, this study provides a key insight for the third level digital divide theory: suggesting that technological agency acts as a key force in reversing traditional resource disadvantages. These findings not only deepen our theoretical understanding of the complex relationship between technology, risk, and human agency but also provide a key practical roadmap for how to empower teachers as cautious and proactive adopters to promote educational equity.

## Electronic Supplementary Material

Below is the link to the electronic supplementary material.

Supplementary Material 1

## Data Availability

The datasets used and/or analyzed during the current study are available from the corresponding author on reasonable request.
