# Supplementary material for: AI literacy and school tier moderate educators’ ethical coping mechanisms through sedative and reverse Matthew effects
Source: Sci Rep. 2026 May 16;16:22239. doi: 10.1038/s41598-026-52376-z (PMC13369881; doi:10.1038/s41598-026-52376-z)
Supplement: Supplementary file 1 — Supplementary Material 1 [file 41598_2026_52376_MOESM1_ESM.docx]

**Supplementary Information**

***Supplementary Table S1***

*Definitions, Operationalization, and Theoretical Foundations of Key Constructs*

| Construct | Theoretical Definition | Operationalization | Theoretical Basis |
| --- | --- | --- | --- |
| Inhibitory Ethical Risk (PI) | It denotes a distinct type of hindrance stressor that mirrors the adverse sociotechnical vision of AI as a threat to the established educational hierarchy. | Assessed as educators' concerns regarding academic integrity, data privacy, and the erosion of critical thinking skills. | [Macro] Sociotechnical Imaginaries^4^  [Micro] Technology Threat Avoidance Theory^25^; Hindrance Stressors^[21](#_ENREF_23)^ |
| Promotional Professional Risk (PP) | It denotes a distinct form of challenge stressor known as progressive vision in which perceived risks are conceptualized as catalysts for professional advancement. | Assessed as the perceived urgency to reform teaching methods and the anxiety of professional obsolescence if AI is not adopted. | [Macro] Sociotechnical Imaginaries^[4](#_ENREF_21)^  [Micro] Transactional Stress Theory - Challenge Appraisal^[7](#_ENREF_8)^ |
| Defensive Ethical Coping (ED) | Fear control oriented tactics designed to limit exposure to ethical threats by employing avoidance behaviors. | Assessed via protective behaviors such as forbidding the use of generative AI tools, implementing tight monitoring, and maintaining dependence on legacy assessment formats. | Extended Parallel Process Model - Fear Control^22^; Technology Threat Avoidance Theory^[25](#_ENREF_27)^ |
| Constructive Ethical Coping (EC) | Danger control oriented behavioral strategies aimed at managing risks through adaptation and proactive integration. | Measured by integrative actions such as redesigning assignments, integrating AI ethics into curriculum, and fostering higher-order thinking. | Extended Parallel Process Model - Danger Control^22^; Transactional Stress Theory - Problem-Focused Coping^[7](#_ENREF_8)^ |
| AI Literacy (AS) | A comprehensive competency structure combining instrumental knowledge and prudent ethical judgment. | Gauged by an objective score derived from a knowledge test covering GenAI mechanisms and applied ethical judgment capabilities. | AI Literacy Framework^9^; AI Literacy for Teachers^[27](#_ENREF_28)^ |

***Supplementary Table S2***

*Full Questionnaire Items, Response Scales, and Sources*

| Construct | Item | Item Text (English & Original Chinese) | Source |
| --- | --- | --- | --- |
| Inhibitory Ethical  Risk (PI) | PI 1 | I am concerned that the unguided use of AI could undermine the atmosphere of fairness in the classroom (e.g., by creating biases or widening the digital divide among students).  我担心缺乏引导的AI使用可能会破坏课堂的公平氛围（例如，产生偏见或加剧学生间的数字鸿沟）。 | Adapted from Schepman & Rodway^[40](#_ENREF_43)^ |
|  | PI 2 | I am concerned that the frequent use of AI tools in teaching could lead to the leakage of my students' or my own personal data and privacy.  我担心在教学中频繁使用AI工具会导致我的学生或我个人的数据及隐私泄露。 | Schepman & Rodway^[40](#_ENREF_43)^ |
|  | PI 3 | I am afraid that students' over reliance on AI to complete assignments could weaken their ability to think independently and solve problems.  我担心学生过度依赖AI完成作业会削弱他们独立思考和解决问题的能力。 | Wang & Wang^[41](#_ENREF_44)^ |
|  | PI 4 | Because the inner workings of AI are unclear to me, I feel uneasy about fully trusting the pedagogical suggestions or factual answers it provides.  因为我不清楚AI的内部运作机制，所以我很难完全信任它提供的教学建议或事实性答案。 | Adapted from Wang & Wang^[41](#_ENREF_44)^ |
| Promotional Professional  Risk (PP) | PP 1 | I believe the rapid development of AI poses a long-term, realistic challenge to the teaching profession, and may even replace some aspects of teaching tasks.  我认为AI的快速发展对教师职业构成了长期且现实的挑战，甚至可能取代教学任务中的某些方面。 | Wang & Wang^[41](#_ENREF_44)^ |
|  | PP 2 | If I do not proactively learn and adapt to AI now, I worry that my teaching methods will become obsolete within the next few years.  如果我现在不主动学习和适应AI，我担心我的教学方法会在未来几年内被淘汰。 | Adapted from Wang & Wang^[41](#_ENREF_44)^ |
|  | PP 3 | I feel a pressure that AI could diminish the core value of teachers in moral education and emotional care, making education more technological and impersonal.  我感到一种压力，AI可能会削弱教师在德育和情感关怀方面的核心价值，使教育变得更加技术化和缺乏人情味。 | Self-developed |
|  | PP 4 | Facing new problems brought by AI (e.g., students cheating), I feel a responsibility to learn how to provide effective educational guidance, but this brings me new pressure.  面对AI带来的新问题（如学生作弊），我觉得有责任学习如何提供有效的教育指导，但这给我带来了新的压力。 | Self-developed |
| Defensive  Coping (ED) | ED 1 | Before using AI-generated teaching materials (e.g., courseware, exercises), I repeatedly verify their accuracy to avoid errors.  在使用AI生成的教学材料（如课件、练习）之前，我会反复核对其准确性以避免错误。 | Adapted from Wang et al.^[42](#_ENREF_45)^ |
|  | ED 2 | For tasks that involve developing students' core competencies or require highly personalized feedback, I tend to limit or avoid using AI.  对于涉及培养学生核心素养或需要高度个性化反馈的任务，我倾向于限制或避免使用AI。 | Self-developed |
|  | ED 3 | When using AI tools in teaching, I proactively inform students and remind them to critically evaluate the AI-generated results.  在教学中使用AI工具时，我会主动告知学生，并提醒他们批判性地评估AI生成的结果。 | Adapted from Wang et al.^[42](#_ENREF_45)^ |
| Constructive  Coping (EC) | EC 1 | I proactively create opportunities in my class to guide students in discussing how to use AI ethically.  我会在课堂上主动创造机会，引导学生讨论如何合乎伦理地使用AI。 | Chiu et al.^[43](#_ENREF_46)^ |
|  | EC 2 | When selecting and using AI teaching tools, I prioritize products that can effectively protect student data and privacy.  在选择和使用AI教学工具时，我优先考虑那些能有效保护学生数据和隐私的产品。 | Chiu et al.^[43](#_ENREF_46)^ |
|  | EC 3 | To respond to challenges posed by AI (such as cheating), I actively reform my assignment and assessment methods (e.g., by increasing formative assessments or project-based learning).  为了应对AI带来的挑战（如作弊），我积极改革我的作业和评估方式（例如，增加形成性评价或项目式学习）。 | Self-developed |
|  | EC 4 | I proactively explore how to leverage AI to provide personalized support for students with diverse learning needs, aiming to promote educational equity in my classroom.  我主动探索如何利用AI为有不同学习需求的学生提供个性化支持，旨在促进我课堂上的教育公平。 | Adapted from Wang et al.^[42](#_ENREF_45)^ |
| AI Literacy (AS) | AS 1 | What is the core feature of Generative AI as we commonly refer to it? (A) To speed up file retrieval. (B) To learn from existing knowledge and create entirely new content. (Correct) (C) To specifically process databases and spreadsheets. (D) To perform real-time translation into multiple languages.  我们通常所说的生成式AI，其核心特征是什么？(A) 加快文件检索速度。(B) 从现有知识中学习并创造全新的内容。(正确) (C) 专门处理数据库和电子表格。(D) 进行实时的多语言翻译。 | Jin et al.^[44](#_ENREF_47)^ |
|  | AS 2 | For current mainstream generative AI, which of the following tasks is it best suited for and most competent at? (A) Accurately predicting stock market trends for the next week. (B) Making fair judgments in complex ethical dilemmas. (C) Diagnosing a very rare disease. (D) Writing a fluent article according to instructions. (Correct)  对于目前主流的生成式AI，以下哪项任务是它最适合且最胜任的？(A) 准确预测下周的股市走势。(B) 在复杂的伦理困境中做出公正的判断。(C) 诊断非常罕见的疾病。(D) 根据指令撰写一篇流畅的文章。(正确) |  |
|  | AS 3 | Assuming you want an AI to help you write a promotional text for school admissions, which of the following approaches is least likely to yield a good result? (A) Telling the AI who the text is written for. (B) Telling the AI the core advantages of the school. (C) Asking the AI to use engaging and persuasive language. (D) Only giving the AI a competitor's admissions brochure. (Correct)  假设你想让AI帮你写一篇学校招生的宣传文案，以下哪种做法最不可能获得好结果？(A) 告诉AI文案是写给谁看的。(B) 告诉AI学校的核心优势。(C) 要求AI使用有吸引力且有说服力的语言。(D) 仅仅丢给AI一份竞争对手的招生简章。(正确) |  |
|  | AS 4 | While using an AI assistant, you find that the admission requirements it provides are from the previous year. What is the most fundamental way to solve this problem? (A) Add a feedback button for outdated information. (B) Periodically update or retrain the AI with the latest materials. (Correct) (C) Transfer all policy-related questions to a human agent. (D) Count the frequency of its incorrect answers.  在使用AI助手时，你发现它提供的招生要求是去年的。解决这个问题最根本的方法是什么？(A) 增加一个对过时信息的反馈按钮。(B) 定期用最新的材料对AI进行更新或重新训练。(正确) (C) 将所有与政策相关的问题转给人工客服。(D) 统计它给出错误答案的频率。 |  |
|  | AS 5 | When using AI for homework or lesson preparation, which of the following attitudes towards the information provided by AI is the most scientific and responsible? (A) Information produced by AI is always credible and can be used directly. (B) AI information is usually more reliable than web pages, but it's best to verify it. (C) AI information is not necessarily credible and must be cross-verified with authoritative sources. (Correct) (D) AI information is generally not credible because it always uses old data.  当利用AI进行备课或布置作业时，对AI提供的信息采取以下哪种态度是最科学负责的？(A) AI产生的信息总是可信的，可以直接使用。(B) AI信息通常比网页更可靠，但最好还是核实一下。(C) AI信息不一定可信，必须与权威信息源进行交叉验证。(正确) (D) AI信息通常不可信，因为它总是使用旧数据。 |  |
|  | AS 6 | Someone says: If you ask AI about news that just happened, its answer is likely to be inaccurate. Is this statement reasonable? (A) Yes, because the AI's knowledge has a cut-off date and it doesn't know about new events that happened after that. (Correct) (B) Yes, because AI is not good at handling numbers. (C) No, because the AI's knowledge is updated in real-time. (D) No, because the AI can automatically connect to the internet.  有人说：如果你问AI刚刚发生的新闻，它的回答很可能是不准确的。这个说法合理吗？(A) 合理，因为AI的知识有截止日期，它不知道那之后发生的新事件。(正确) (B) 合理，因为AI不擅长处理数字。(C) 不合理，因为AI的知识是实时更新的。(D) 不合理，因为AI可以自动联网。 |  |
|  | AS 7 | If a school uses AI to screen teacher resumes, which of the following serious ethical risks is most likely to occur? (A) The AI might not understand the personal honors listed in the resume. (B) The AI might misjudge based on differences in resume layout. (C) The AI might not handle English resumes well. (D) The AI might replicate hidden biases present in past recruitment. (Correct)  如果学校使用AI来筛选教师简历，最有可能出现以下哪种严重的伦理风险？(A) AI可能看不懂简历上列出的个人荣誉。(B) AI可能因为简历排版格式的不同而产生误判。(C) AI可能处理不好英文简历。(D) AI可能会复制过往招聘中存在的隐性偏见。(正确) |  |
|  | AS 8 | Someone believes: The content we input when chatting with an AI is absolutely secure, and there's no need to worry about privacy leaks. What do you think of this view? (A) It's correct because the content is encrypted during transmission. (B) It's correct because AI is a black box and won't leak information. (C) It's incorrect because the AI might remember and inadvertently leak sensitive information during its training. (Correct) (D) It's incorrect because hackers can easily crack the encryption.  有人认为：我们在与AI聊天时输入的内容是绝对安全的，不用担心隐私泄露。你如何看待这种观点？(A) 正确，因为内容在传输过程中是加密的。(B) 正确，因为AI是一个黑箱，不会泄露信息。(C) 错误，因为AI可能会记住并在其训练中无意间泄露敏感信息。(正确) (D) 错误，因为黑客很容易破解加密。 |  |
| *Note*. Full citations for all sources are provided in the References section. Adapted from indicates that the original scale was modified to fit the current research context. Unless otherwise specified, items for PI, PP, ED, and EC were rated on a 7 point Likert scale (1 = Strongly Disagree, 7 = Strongly Agree). AS (AI Literacy) was evaluated using an objective knowledge test (Range: 0–8), where the final value represents the total sum of correct answers. | | | |

***Supplementary Table S3***

***AI Literacy (AS) Instrument items and statistics***

| Item | Content Domain / Abbreviated Question | Difficulty (Correct %) | Discrimination Index |
| --- | --- | --- | --- |
| AS 1 | GenAI Features: Understanding the predictive mechanism of generative AI | 56.80% | 0.19 |
| AS 2 | Capabilities: Identifying tasks that current AI cannot perform accurately | 73.70% | 0.35 |
| AS 3 | Prompting: Selecting effective prompt engineering strategies | 54.40% | 0.39 |
| AS 4 | Model Updating: Understanding the necessity of retraining or updating data to fix outdated information. | 63.80% | 0.19 |
| AS 5 | Skepticism: Critical evaluation of AI-generated educational advice | 54.60% | 0.34 |
| AS 6 | Knowledge Cut-off: Understanding that AI knowledge is limited by training data dates | 62.80% | 0.14 |
| AS 7 | Ethical Bias: Identifying potential algorithmic biases in recruitment | 52.99% | 0.40 |
| AS 8 | Privacy Risks: Understanding risks of data leakage in AI conversations | 74.10% | 0.47 |
| Note: *N* = 502. Items were scored dichotomously (1 = correct, 0 = incorrect). Difficulty represents the percentage of correct responses. Discrimination refers to the corrected item total correlation. The total test showed moderate difficulty (*M* = 4.93/8, *SD* = 1.98) and acceptable reliability (*KR*-20 = 0.605) for a broad spectrum formative index. Lower discrimination indices on specific factual items (e.g., AS 1, AS 6) are expected given the instrument's coverage of diverse, orthogonal knowledge domains (e.g., technical mechanisms vs. ethical norms). | | | |
